# MET expression during prostate cancer progression

**DOI:** 10.18632/oncotarget.8829

**Published:** 2016-04-18

**Authors:** Esther I. Verhoef, Kimberley Kolijn, Maria J. De Herdt, Berdine van der Steen, A. Marije Hoogland, Hein F.B.M. Sleddens, Leendert H.J. Looijenga, Geert J.L.H. van Leenders

**Affiliations:** ^1^ Department of Pathology, Erasmus Medical Centre, Rotterdam, The Netherlands; ^2^ Othorhinolaryngology and Head and Neck Surgery, Erasmus Medical Centre, Rotterdam, The Netherlands

**Keywords:** MET, prostate cancer, progression, protein

## Abstract

Tyrosine-kinase inhibitors of the hepatocyte growth factor receptor MET are under investigation for the treatment of hormone-refractory prostate cancer (HRPC) metastasis. Analysis of MET protein expression and genetic alterations might contribute to therapeutic stratification of prostate cancer patients. Our objective was to investigate MET on protein, DNA and RNA level in clinical prostate cancer at various stages of progression.

Expression of MET was analyzed in hormone-naive primary prostate cancers (N=481), lymph node (N=40) and bone (N=8) metastases, as well as HRPC (N=54) and bone metastases (N=15). MET protein expression was analyzed by immunohistochemistry (D1C2 C-terminal antibody). MET mRNA levels and *MET* DNA copy numbers were determined by *in situ* hybridization.

None of the hormone-naive primary prostate cancer or lymph node metastases demonstrated MET protein or mRNA expression. In contrast, MET protein was expressed in 12/52 (23%) evaluable HRPC resections. RNA *in situ* demonstrated cytoplasmic signals in 14/54 (26%) of the HRPC patients, and was associated with MET protein expression (p=0.025, χ^2^), in absence of *MET* amplification or polysomy. MET protein expression was present in 7/8 (88%) hormone-naive and 10/15 (67%) HRPC bone metastases, without association of HRPC (p=0.37; χ^2^), with *MET* polysomy in 8/13 (61%) evaluable cases.

In conclusion, MET was almost exclusively expressed in HRPC and prostate cancer bone metastasis, but was not related to *MET* amplification or polysomy. Evaluation of MET status could be relevant for therapeutic stratification of late stage prostate cancer.

## INTRODUCTION

The tyrosine-kinase receptor MET and its ligand hepatocyte growth factor (HGF) play important roles in stromal-epithelial interactions in a diversity of tissues. Upon secretion by mesenchymal cells HGF targets the MET receptor, contributing to embryogenesis, tissue development, proliferation and differentiation [1–3]. Over-expression and hyper-activation of MET has been found in various cancer types and is often associated with poor outcome, or a role in development and metastasis of cancer [4–9]. Previous studies have shown that MET expression occurs predominantly in pre-existent basal and intermediate prostate glandular epithelium [9–11]. In prostate cancer MET is predominantly expressed in cells with an intermediate phenotype and enhanced at the tumor perimeter. Activation of MET in prostate cancer cell line DU145 results in cell migration, invasion and the acquisition of a stem-like phenotype [10–12].

Hormone-deprivation is the first choice of therapy for metastasized prostate cancer. Most patients, however, suffer from hormone-refractory prostate cancer (HRPC) within a few years after initial treatment. Tyrosine-kinase inhibitor Cabozantinib, which targets both MET and VEGFR2, is currently under investigation for its effects on metastasized prostate cancer [13]. A phase II trial demonstrated efficacy of Cabozantinib on bone scan lesions and reduction of soft tissue tumor load in HRPC patients [14–17]. In order to determine whether individual patients could be stratified for therapy, it is important to gain insight in MET protein expression in prostate cancer. By detailed analysis of five commercially available MET antibodies, De Herdt *et al*. have recently shown that clone D1C2 was highly specific for the C-terminus of the MET receptor, while the other antibodies demonstrated less sensitive or non-specific behavior [18]. The aim of this study was to investigate MET on protein, DNA and RNA level in clinical prostate cancer at various stages of progression.

## RESULTS

### MET expression in hormone-naive prostate cancer

**Figure 1:**
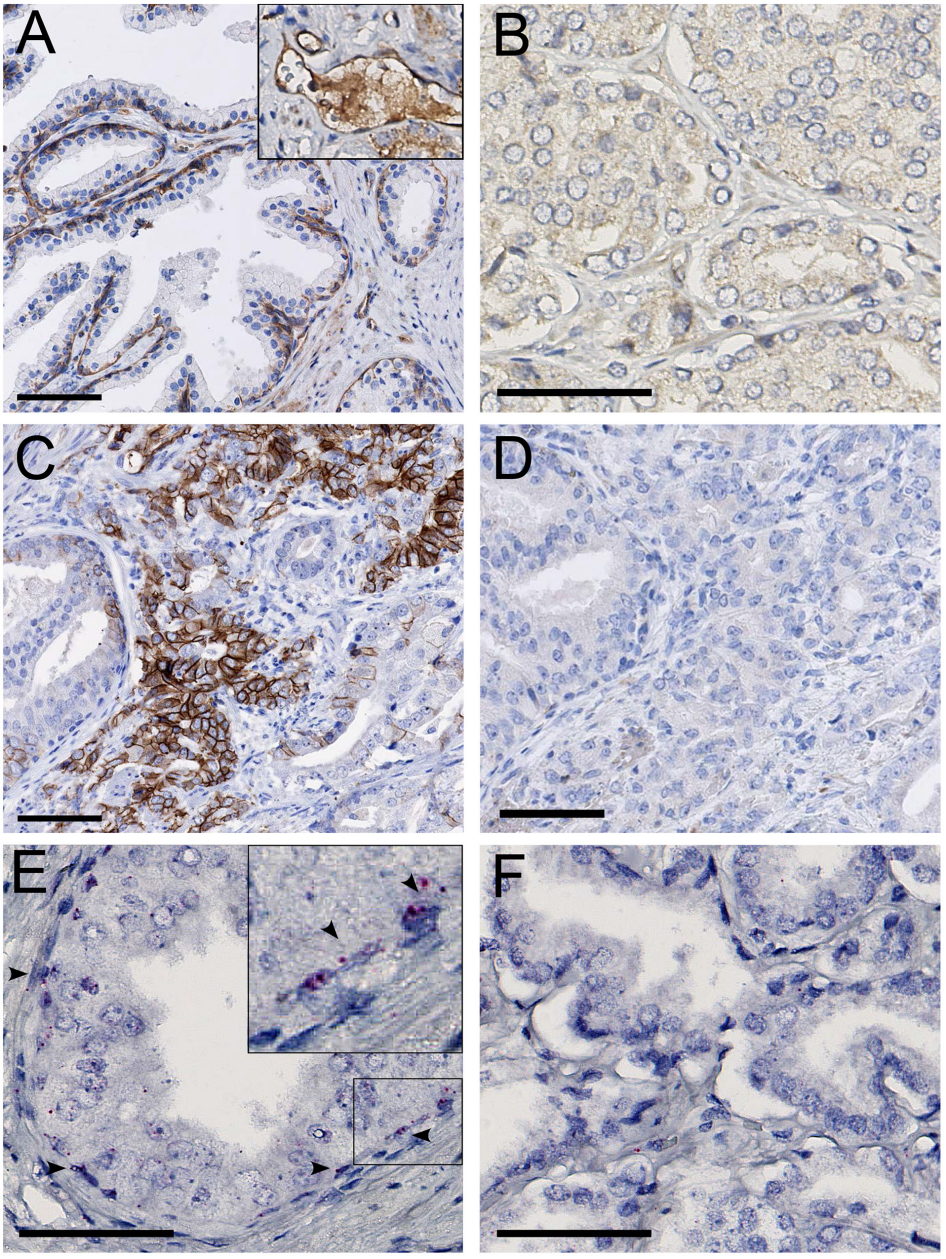
**A.** MET protein staining in basal epithelial and endothelial cells (*inset*), in the normal prostate. **B.** MET protein absence in localized prostate cancer. **C.** N-cadherin positive area in localized prostate cancer. **D.** MET protein absence in the N-cadherin positive area. **E.** MET RNA expression in normal prostate basal epithelial cells (*arrowheads*). **F.** Absence of MET RNA in localized prostate cancer. Scale bars represent 50 μm at 20x (A, C, D) or 40x (B, E, F) magnification.

MET protein expression was observed in pre-existent basal and atrophic luminal glandular epithelium, which served as internal positive controls, and was variably expressed in both normal and tumor-associated endothelial cells (Figure 1A). None of the 481 hormone-naive prostate cancer samples revealed MET expression (Figure 1B). To verify these results, we additionally analyzed MET in 25 whole sections of prostate cancer at radical prostatectomy containing various tumor growth patterns, which also proved MET protein negative despite positive internal controls. To exclude MET expression in rare tumor areas undergoing E/N-cadherin switching indicative for epithelial-mesenchymal transition, we determined MET expression in consecutive tissue slides of independent N-cadherin positive tumor areas, which likewise did not demonstrate MET staining (Figure 1C, 1D). MET RNA *in situ* hybridization signals were present in basal and atrophic luminal cells of normal glands serving as positive controls (Figure 1E). RNA signals were not observed in any of the samples (Figure 1F). Since neither MET protein nor RNA was observed, we did not perform DNA *in situ* hybridization.

### MET expression in HRPC

**Figure 2:**
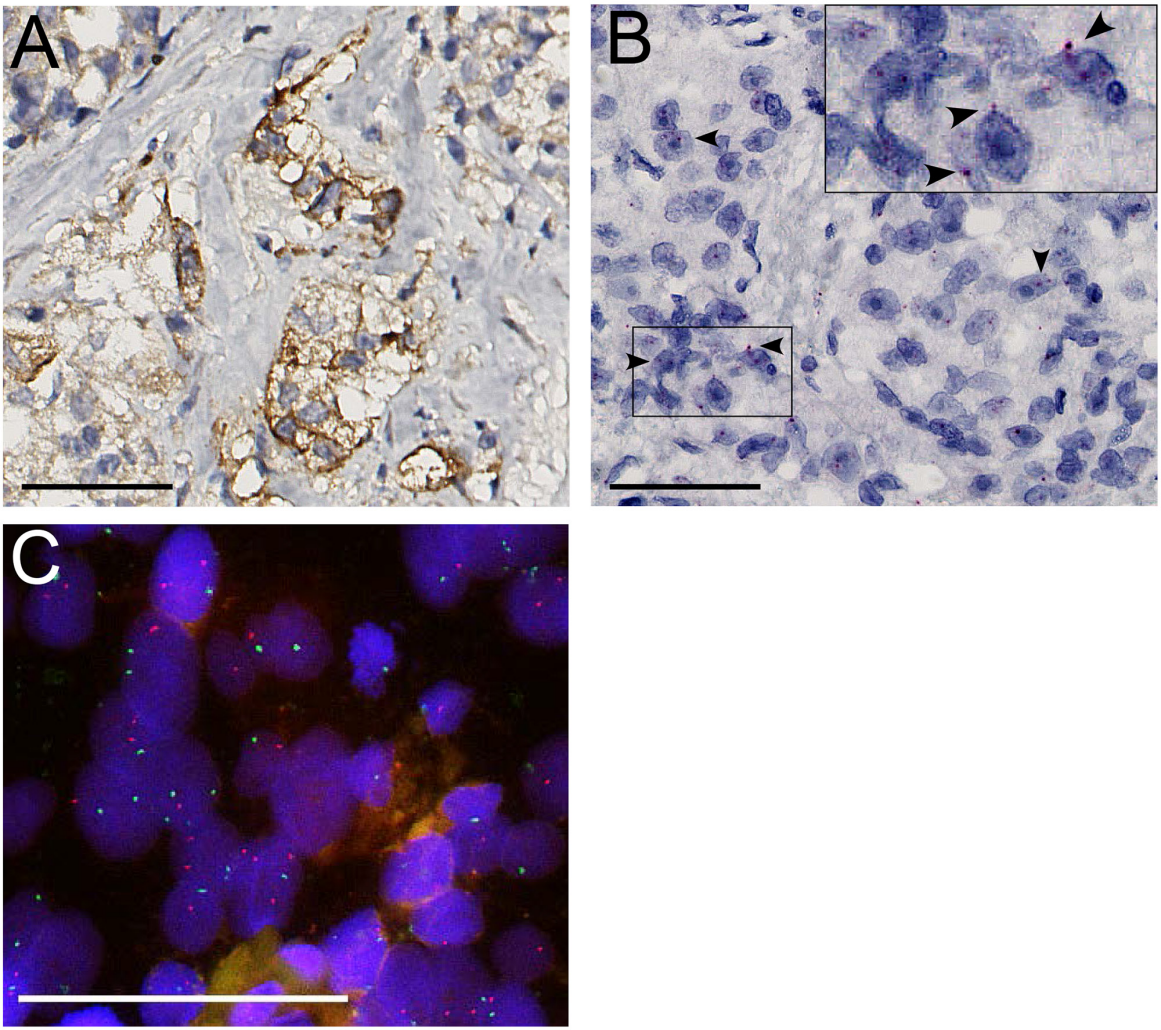
**A.** MET protein expression. **B.** Positive RNA *in situ* signals (*arrowheads*). **C.** FISH did not reveal polysomy or *MET* amplification (*MET* red, *SE7* green). Scale bars represent 50 μm at 40x (A, B) or 63x (C) magnification.

Membranous MET protein expression ranged from 1% to 20% and was found in 12/52 evaluable (23%) HRPC patients at palliative trans-urethral resection as compared to 1 of 49 (2%) hormone-naive controls (p=0.002, χ^2^) (Figure 2A). A total of 1-5% tumor cells were positive in 7 cases, 5-10% in 4 cases and 10-20% in 1 case. RNA *in situ* demonstrated cytoplasmic signals in 14/54 (26%) HRPC patients and 4/50 (12%) hormone-naive prostate cancer cases. Although there was a trend of elevated RNA in HRPC, this association was not significant (p=0.085, χ^2^) (Figure 2B). Presence of RNA *in situ* signals was associated with MET protein expression in 6/21 (28%) positive samples out of 97 evaluable cores (p=0.025, χ^2^). FISH did not reveal *MET* amplification or polysomy in any of the cases (Figure 2C).

### MET expression in prostate cancer metastasis

**Figure 3:**
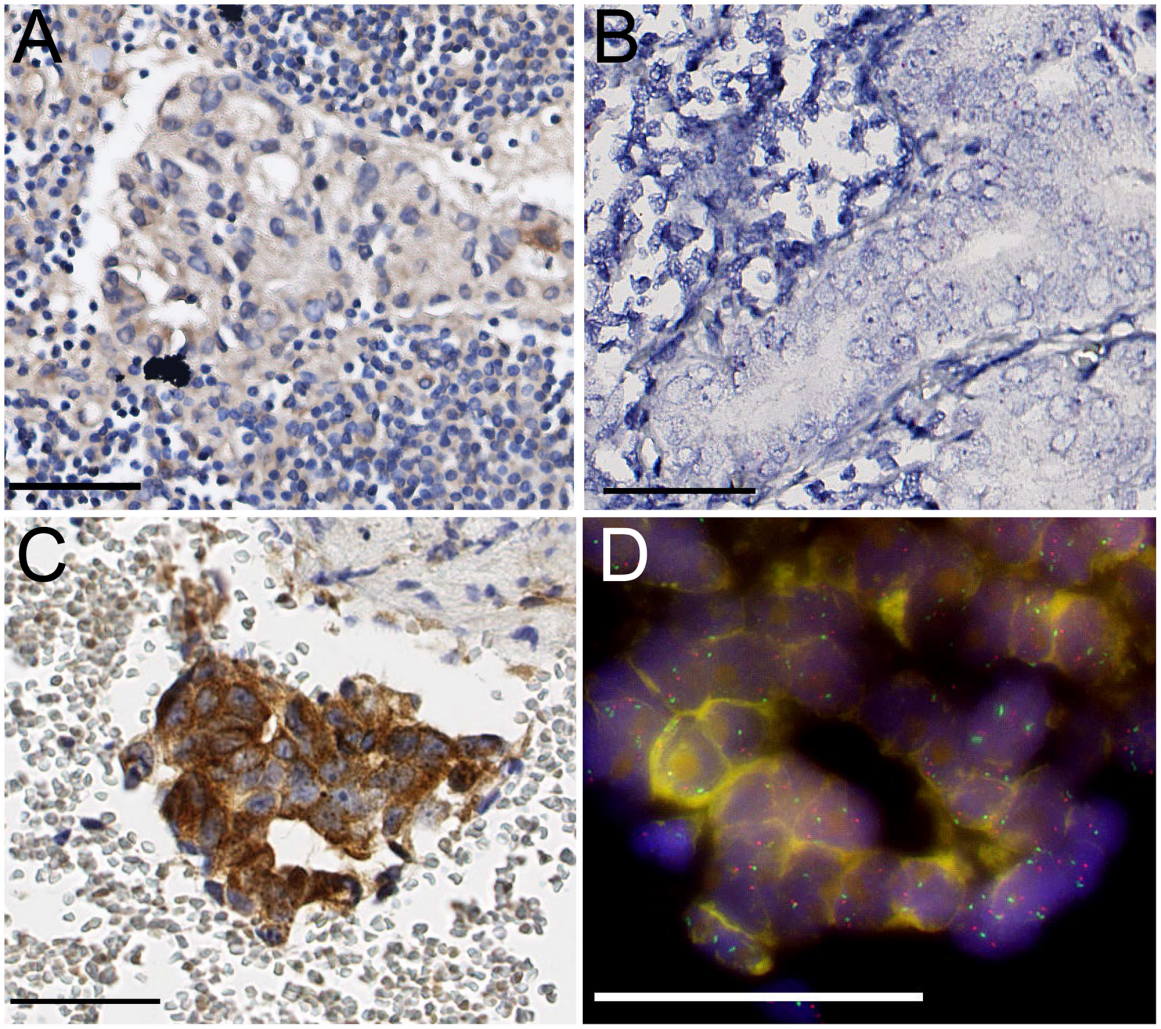
**A.** Absence of specific MET protein expression in hormone-naive lymph node metastasis. **B.** Absence of RNA *in situ* signals in lymph node metastasis. **C.** Strong MET protein expression in HRPC bone metastasis. **D.** Chromosome 7 polysomy in HRPC bone metastasis (*MET* red, *SE7* green). Scale bars represent 50 μm at 40x (A, B, C) or 63x (D) magnification.

None of the 40 evaluable hormone-naive lymph node metastases demonstrated expression of MET protein or RNA (Figure 3A, 3B). FISH for *MET* did not reveal amplification or polysomy in any of the cases. In contrast, MET immunohistochemistry revealed expression in 20% up to 100% of tumor cells in 18/23 (78%) prostate cancer bone metastasis (Figure 3C). MET protein expression was present in 7/8 (88%) hormone-naive and 10/15 (67%) HRPC bone metastases, and was not associated with HRPC (p=0.37; χ^2^). RNA *in situ* hybridization was not feasible on bone metastasis due to RNA degradation during decalcification of bone tissue using formic acid. [19] Polysomy of *MET* was found in 8/13 (61%) evaluable cases, with an average of 2.6 copy numbers per nucleus (range 2.2-3.3), but was not associated with HRPC (p=0.293, χ^2^) (Figure 3D). None of the samples demonstrated amplification. *MET* polysomy status was not associated with MET protein (p=0.51, χ^2^).

## DISCUSSION

Recent phase II and III studies have demonstrated that treatment of metastasized HRPC patients with Cabozantinib led to reduced tumor load on bone scans and in soft tissues together with prolonged progression-free survival [15]. To determine whether subpopulations of prostate cancer patients could be identified for therapeutic stratification, we evaluated MET expression at clinically relevant stages of disease progression. C-terminal MET protein expression was neither found in hormone-naive primary prostate cancer nor lymph node metastasis. In contrast, C-terminal MET protein expression was present in HRPC in 23% of palliative transurethral resection specimens and 72% of bone metastases, but was not related to *MET* polysomy or amplification.

Various *in vitro* and *in vivo* studies have shown the involvement of MET in development and metastasis of prostate cancer [20–22]. Activation of the HGF/MET axis in prostate cancer cell lines resulted in migration and induced orthotopic tumor formation [4, 10, 12]. These effects went together with the induction of a stem-like phenotype suggesting a relation between epithelial-mesenchymal transition and stem cell biology [12, 23, 24]. While many groups have studied the role of HGF/MET signaling in epithelial-mesenchymal transition, little is known of the actual role of this pathway in clinical prostate cancer [25, 26]. Results of MET protein expression in clinical prostate cancer are variable due to use of different antibodies and staining techniques. De Herdt *et al*. have recently investigated the reliability of five commercially available antibodies targeting MET and found that C-terminal clone D1C2 showed the highest sensitivity and membranous specificity for MET on formalin-fixed paraffin-embedded tissues [18]. We were not able to demonstrate D1C2 labelling in hormone-naive prostate cancer in a large set primary prostate cancer cases [27]. Staining of serial sections did not reveal MET expression in N-cadherin positive areas. Since we have previously shown that N-cadherin is the most representative marker for epithelial-mesenchymal transition in clinical prostate cancer, these findings indicate that the HGF/MET pathway plays a minor role in early hormone-naive prostate cancer [28].

Albeit low to absent in hormone-naive prostate cancer, MET expression was found in HRPC specimens and bone metastases. These findings are in line with previous reports showing enhanced MET expression in HRPC bone metastases [4, 14]. This over-expression of MET was not related to genetic polysomy or amplification, which has also been found by Jardim *et al*. in a set of 40 metastatic prostate cancers [29]. The induction of MET in HRPC could be explained by disruption of normal androgen receptor (AR) signaling, since this pathway has a repressive effect on MET [30, 31]. During hormone-deprivation therapy, the repressive effect could be abolished leading to up-regulation of MET [10]. While MET was over-expressed in bone metastases, there was no statistically significant difference between hormone-naive and -refractory prostate cancer. Clonal selection of primary tumors might play a role in development of metastasis to bone, since Chu *et al*. found that abrogation of RANK, c-MYC or MET strongly reduced bone metastasis *in vivo* [32]. Additionally, the bone micro-environment might contribute to over-expression of MET. Cytokines such as TGFβ, PDGF and IGF1 are actively secreted by the bone micro-environment, and osteoblasts are known to secrete HGF ligand [33, 34].

Despite improvement on bone scans and secondary side effects such as progression-free survival, treatment with Cabozantinib did not lead to better overall survival of HRPC patients [15, 35]. In order to explain the clinical results, Varkaris *et al*. performed functional studies on patient-derived xenografts and Cabozantinib-treated patients [36]. While Cabozantinib treatment initially led to reduced phosphorylation, pMET levels normalized after 9 days and presence of MET was not required for tumor growth. On the other hand, inhibition of MET and VEGFR2 activity in tumor associated endothelial cells resulted in sustained growth inhibition. Finally, up-regulation of tumor FGFR1 expression and adaptation of bone micro-environment putatively contributed to therapy-resistance. Despite the lack of increase in overall survival of Cabozantinib alone, its combination with other treatment modalities such as Abiraterone could potentially optimize treatment outcomes [36–38].

The strong points of this study include the thorough preceding characterization of the D1C2 antibody and the use of a wide range of clinically relevant prostate cancer specimens at various stages of disease progression. Disadvantages of the current study are that MET immunohistochemistry was scored visually and not by automated image-analysis. In addition, MET protein expression levels were determined by chromogenic detection at paraffin-embedded tissues, which might reach insufficient sensitivity to detect very low levels of protein.

In conclusion, we demonstrate that MET is highly expressed in HRPC at palliative transurethral resection specimens and bone metastases, while expression is low to absent in hormone-naive primary prostate cancer and lymph node metastases. Protein expression of MET was not related to genetic polysomy or amplification, but was putatively caused by modifications in micro-environmental and tumor signaling pathways. Evaluation of MET status could be relevant for therapeutic stratification of late stage prostate cancer.

## MATERIALS AND METHODS

### Clinical specimens

Four prostate cancer cohorts representing various disease stages were used in this study. The first cohort consisted of 481 hormone-naive prostate cancer patients who had undergone radical prostatectomy for their disease. All patients had been diagnosed with prostate cancer in the scope of the European Randomized Study of Screening for Prostate Cancer, Rotterdam section [39, 40]. As described previously, a tissue micro-array (TMA) was constructed including three representative cores of each radical prostatectomy [27]. In addition, whole tissue slides of 25 radical prostatectomy specimens with hormone-naive prostate cancer were used as control as well as consecutive sections of five selected radical prostatectomy specimens containing hormone-naive prostate cancer with expression of membranous N-cadherin, as marker for epithelial-mesenchymal transition [28]. Secondly, a TMA of palliative transurethral resections from 64 HRPC patients treated for urinary obstruction between 1995 and 2009, together with 54 hormone-naive controls from radical prostatectomies and transurethral resections, was constructed. Three representative tissue cores per sample were included in the TMA. A third TMA consisted of 40 hormone-naive lymph node metastases. The fourth cohort consisted of whole tissue sections of 8 hormone-naive and 15 HRPC bone metastases. Use of tissue samples was approved by the Erasmus Medical Centre Medical Ethics Committee (MEC-2011-295).

### Immunohistochemistry

Briefly, 5 μm formalin-fixed, paraffin-embedded sections were dewaxed and rehydrated using xylene and ethanol, and endogenous peroxidase was blocked for 20 minutes in 0.3% H_2_O_2_ in PBS. Heat-induced antigen retrieval was done in TRIS-EDTA buffer (pH=9; Klinipath, Duiven, The Netherlands) using a pressure cooker at 1.2 bar. MET antibody (clone D1C2; Cell Signalling, Leiden, The Netherlands) diluted 1:100 in 0.2% PBS/ BSA was incubated overnight at 4°C. Antibody was visualized using Vectastain ABC (PK-6100, Brunschwig Chemie, Amsterdam, The Netherlands) and Envision (K500711, DAKO, Heverlee, Belgium), followed by counterstaining with hematoxylin. Basal and luminal atrophic epithelial cells from normal prostate glands served as internal control [11].

N-cadherin staining was done on five selected paraffin-embedded sections, of which a consecutive section was stained for MET [28]. Sections were dewaxed and rehydrated using xylene and ethanol, and endogenous peroxidase was blocked for 20 minutes in 0.3% H_2_O_2_ in PBS. Heat-induced antigen retrieval was done in citrate buffer (pH 6.0; Sigma-Aldrich, St. Louis, USA) for 15 minutes. Mouse anti-N-cadherin was diluted 1:50 (clone 6G11; DAKO) in 1% PBS/ BSA and incubated overnight at 4°C. Antibody was visualized using Envision (DAKO) followed by counterstaining with hematoxylin.

### RNA *in situ* hybridization

RNA *in situ* hybridization on formalin-fixed paraffin-embedded tissue was done using RNAscope (ACD Bio, Hayward, USA). Fresh 5 μm sections were heated at 60°C for 30 minutes, deparaffinized and rehydrated. After blocking of endogenous peroxidase for 15 minutes, slides were heated at 102°C for 15 minutes and treated with protease (#310020, ACD Bio) for 15 minutes. The Hs-cMET specific target probe provided by the manufacturer (#310051, ACD Bio), targeting base pairs 1236-2257 of MET, was hybridized for 2 hours. Signal amplification on the probe was followed by visualization with fast-red according to manufacturer's protocol and counterstained with hematoxylin. Probes for housekeeping gene ubiquitin C and bacterial gene dapB served as positive and negative control respectively (#310041 and #310043, ACD Bio). Basal and luminal atrophic epithelial cells from normal prostate glands served as internal control. Cells were defined positive when two or more ISH signals per cell were present.

### DNA *in situ* hybridization

To establish *MET* copy number, fluorescence *in situ* hybridization (FISH) was done using a commercial probe targeting MET (KBI-10719, Kreatech, Amsterdam, The Netherlands). An *SE7* centromere probe served as copy number control. Tissue sections were dewaxed and rehydrated, followed by heating in citrate buffer (CB999, Klinipath) for 13 minutes. Tissue was treated with pepsine (P7000-25G, Sigma-Aldrich) for 20 minutes and dehydrated, followed by probe hybridising overnight at 37°C. Copy numbers were calculated as an average of probe hybridization in 20 nuclei of prostate cancer cells. A *MET/SE7* ratio of 1 with more than 2 signals per nucleus was defined as *MET* polysomy, while a ratio of >1 with presence of more than 2.2 *MET* signals per nucleus was considered amplification.

### Statistics

MET expression in both hormone-naive prostate cancer and HRPC was compared using Pearson's Chi-square (X^2^) test. Two-sided p-values of <0.05 were considered statistically significant. Statistical analysis was done with the Statistical Package for Social Sciences (SPSS version 21, IBM, Chicago, USA).
